# Protocol for a systematic review of the effects of interventions for vaccine stock management

**DOI:** 10.1186/s13643-018-0922-3

**Published:** 2019-01-08

**Authors:** Chinwe Juliana Iwu, Anelisa Jaca, Leila H. Abdullahi, Ntombenhle Judith Ngcobo, Charles Shey Wiysonge

**Affiliations:** 1 Division of Health Systems and Public Health, Department of Global Health, Faculty of Medicine and Health Sciences, Stellenbosch University, Cape Town, South Africa; 2 Cochrane South Africa, South African Medical Research Council, PO Box 19070, Tygerberg, Cape Town, 7505 South Africa; 3 Save the Children International (SCI), Somalia/Somaliland Country Office, Nairobi, Kenya; 4 Department of Paediatrics, Red Cross War Memorial Children’s Hospital, University of Cape Town, Cape Town, South Africa; 5 Division of Epidemiology and Biostatistics, School of Public Health and Family Medicine, University of Cape Town, Cape Town, South Africa

**Keywords:** Vaccine stock management, Vaccine stock-outs, Vaccine availability, Vaccine supply chain, Vaccine shortages, Vaccine inventory management

## Abstract

**Background:**

Inadequate vaccine stock management in health facilities leads to vaccine stock-outs. The latter threatens the success of immunisation programmes. Countries have used various approaches to reduce stock-outs and improve vaccine availability, but we are not aware of a systematic review of these interventions. This protocol describes the methods we will use to assess the effects of existing approaches for improving vaccine stock management.

**Methods:**

We include randomised and non-randomised studies identified through a compehensive search of peer-reviewed and grey literature databases. We will search PubMed, Cochrane Central Register of Controlled Trials, Embase, Web of Science, PDQ-Evidence and Scopus. We will also search websites of the World Health Organisation (WHO), Global Alliance for Vaccine and Immunisation, PATH Vaccine Resources Library and United Nations Children’s Fund. In addition, we will search the WHO International Clinical Trials Registry Platform and reference lists of included studies and relevant reviews. Finally, we plan to do a citation search for included studies. We will use Cochrane recommended methods to screen search outputs, assess study eligibility and risk of bias, extract and analyse study results. We will use the Grading of Recommendations Assessment, Development and Evaluation (GRADE) tool to assess the certainty of the evidence on the effects of the interventions.

**Discussion:**

We believe that the findings of this review will serve as valuable information for policy makers on ways to improve vaccine stock management and vaccine availability. When vaccine availability is improved, those who need them, especially children, will be adequately protected from vaccine-preventable diseases.

**Systematic review registration:**

PROSPERO CRD42018092215

**Electronic supplementary material:**

The online version of this article (10.1186/s13643-018-0922-3) contains supplementary material, which is available to authorized users.

## Background

The success of immunisation programmes depends on a well-functioning supply chain that ensures the constant availability of quality vaccines to the target population [1–3]. Effective vaccine stock management is one of the criteria for an effective vaccine supplychain [1, 4]. Vaccine stock management at health facility level involves the checking and monitoring of vaccines on arrival at a storage point, during storage and when they are administered to the users [1, 2]. Adequate vaccine stock management helps to maintain the quality of vaccines [1, 2] and prevent vaccine stock-outs. Vaccine stock-outs refer to the absence of vaccine(s) at the point of service delivery to the patient [3, 5]. An analysis of global data on effective vaccine management assessments between 2009 and 2014 showed that most low- and middle-income countries performed below the minimum standard for adequate vaccine stock management [6].

Recent data reported by countries in World Health Organisation (WHO) and United Nations Children’s Fund (UNICEF) joint reporting show that each year at least one-third of countries experience one or more vaccine stock-outs lasting for at least 1 month [7]. The most vulnerable groups who suffer the effects of vaccine stock-outs in resource-constrained settings are the urban poor and rural communities who depend on public facilities for health services. When vaccines are not available, these recipients of public health services are obliged to make repeated and costly trips to health facilities. Ultimately, immunisation targets are not met, universal health coverage remains an elusive dream and lives are lost [8].

Due to the upward trend in the rates of vaccine stock-outs, countries are currently creating approaches to improve vaccine stock management [7, 9]. The approaches for improving vaccine availability may include the use of digital systems to monitor vaccine stock levels in real time [10–12] . These dashboards measure performance and make them visible for managers to make informed decisions [13]. Another vaccine stock management approach involves the crowd sourcing of reports of stock-outs from patients and community volunteers. These reports are then sent to relevant health system structures to elicit system changes for improving vaccine availability [14]. However, we are not aware of a systematic review of these and other potential interventions for improving vaccine stock management.

### Objective

We aim to assess the effects of approaches used for vaccine stock management at facility level.

## Methods

This systematic review protocol has been prepared according to the Preferred Reporting Items for Systematic Reviews and Meta-Analyses Protocol (PRISMA-P) 2015 guideline (Additional file 1).

### Registration of the review

We registered the systematic review in the International Prospective Register of Systematic Reviews (PROSPERO) [15].

### Eligibility criteria for studies

We will include individually randomised trials, cluster randomised trials, controlled before-after studies, interrupted time series studies and repeated cross-sectional studies. Eligible participants include the healthcare systems which deliver vaccines, healthcare facilities where vaccines are administered, healthcare workers involved in providing immunisation services and recipients of immunisation services.

We plan to include interventions targeting recipients or providers of immunisation services. Recipient-oriented interventions may include the involvement of end-users in monitoring vaccine availability at facilities, e.g. using mobile phone services or hotline platforms. Examples of interventions directed at providers of immunisation services include education or training, audit and feedback, prompts or reminders and supportive supervision. We will also include interventions targeting the health system offering immunisation services, e.g. action plans, re-designing (components) of the supply chain and integration with other services. Other interventions intended to ensure vaccine availability, including multi-component interventions, are also eligible for inclusion. We will consider the following as eligible comparisons: standard vaccine stock management practices in the study setting, alternative interventions and similar interventions implemented with different degrees of intensity.

Our primary outcomes are vaccine availability and vaccine stock-outs. We will measure vaccine availability as the proportion of vaccination days in which vaccines were available and no one eligible for vaccination was turned back for lack of vaccines; but will also consider other measures of vaccine availability used by the authors of included studies. Vaccine stock-out rates in the review will be measured as the percentage of facilities that experienced a stock-out of a specific vaccine that the site is expected to provide, at any point, within a defined period; or other definitions as used in included studies. Our secondary outcomes include acceptability, adverse events and cost of the intervention, as well as other outcomes as reported by included studies.

### Data sources

We will develop a comprehensive search strategy for both peer-reviewed and grey literature. We will search the following databases PubMed, Cochrane Central Register of Controlled Trials (CENTRAL), Embase, WHO Library Information System (WHOLIS), Web of Science, PDQ (Pretty Darn Quick)-Evidence and Scopus. We will also search the websites of WHO, Global Alliance for Vaccine and Immunisation, PATH Vaccine Resources Library and UNICEF. In addition, we will search the WHO International Clinical Trials Registry Platform, reference lists of included studies and related systematic reviews and citations of included studies. A preliminary search strategy developed for PubMed is found in the Appendix.

### Data collection and analyses

Two authors will independently screen the titles and summaries of records retrieved from the search for potentially eligible studies. We will obtain full-texts for all the potentially eligible studies. Two authors will assess these full-text publications for eligibility. Any disagreements between the two authors regarding study eligibility will be resolved by discussion and consensus. A third author will arbitrate any unresolved disagreements. We will provide a table with the characteristics of the included studies, and another of excluded studies with reasons for their exclusion. We will seek additional information, for studies with missing information, to assist us in our decision-making process.

For each included study, two authors will independently extract information using a piloted data extraction form. Extracted data will include study design, participant, intervention and outcome characteristics as well as outcome data. Any differences will be resolved through discussion and consensus. A third author will be consulted to arbitrate if disagreements persist between the two authors. If there are missing data, we will contact study investigators to obtain the missing information. Two authors will independently assess risk of bias in included studies using the appropriate tool for randomised trials [16] and non-randomised studies [17]. Differences in judgement will be resolved by discussion and consensus, with arbitration by a third author.

We will present study results as risk ratios for dichotomous data (e.g. frequency of vaccine stock-outs), and mean differences for continuous data (e.g. duration of vaccine stock-outs) will be presented as mean difference. We will combine data from clinically homogenous studies (in terms of designs, participants, interventions and outcomes) using random-effects meta-analysis. However, if we come across variation between studies, the findings will be summarised in a narrative format. We will analyse results of interrupted time series studies using regression analysis with time trends before and after the interventions [18, 19]. We will assess certainty of the evidence of effects of interventions using the Grading of Recommendations, Assessment, Development and Evaluation (GRADE) approach [16].

We will look out for and correct any errors made in the analysis of included studies. For example, if clustering is not addressed in an eligible cluster randomised trial, we will re-analyse the data if sufficient information is available. Otherwise, we will request necessary data from the authors or attempt to adjust data for clustering by inflating the standard errors by multiplying them by the square root of the design effect [16]. The adjusted effect will then be added in the meta-analysis. We will assess statistical heterogeneity among study results using the *I*^2^ statistic. We will consider heterogeneity as substantial, if the *I*^2^ is 50% or more. We will investigate the causes of substantial statistical heterogeneity using subgroup analyses. We will define subgroups based on participant and study design characteristics. We will use the chi-squared test for subgroup differences to assess for subgroup interactions. We will carry out sensitivity analyses, if applicable on aspects that could potentially affect the meta-analysis results such as study designs and overall risk of bias. We may also conduct a sensitivity analysis to explore the effects of fixed- versus random-effects analyses for outcomes with statistical heterogeneity [16].

## Discussion

This systematic review will examine the effectiveness of existing approaches for managing vaccine stock levels at health facilities, in order to prevent vaccine stock-outs. Study findings will serve as valuable information for policy makers on ways to improve vaccine stock management and vaccine availability. When vaccine availability is improved, target populations will be adequately protected from vaccine-preventable diseases. Furthermore, there will be reduction in the number repeated visits that patients should make in a bid to get vaccinated. This will increase their trust in the health systems. Ultimately, there will be a reduction in deaths caused by vaccine-preventable diseases as well as an improvement in other health outcomes.

### Additional file

Additional file 1: PRISMA P checklist for the protocol. (DOCX 21 kb)

## Appendix

**Table 1:** Search strategy for PubMed

Search	Query	Items found
7	Search (#1 OR #2 OR #12 OR 4 OR #5) AND #6	83
6	Search (low-income countries) OR (middle-income countries)	28798
5	Search (vaccines OR Vaccine) AND “Supply and distribution”	2507
4	Search (Vaccine OR Vaccines) AND “Supply chain Management”	15
3	Search “Vaccine stockout” OR “Vaccines stockout”	3
2	Search drug storage AND (Vaccine OR Vaccines)	994
1	Search “Vaccine stock management” OR “Vaccine Management”	131

## Abbreviations

CENTRAL: Cochrane Central Register of Controlled Trials
GRADE: Grading of Recommendations Assessment, Development and Evaluation
GVAP: Global Vaccine Action Plan
ICTRP: International Clinical Trials Registry Platform
UNICEF: United Nations Children’s Fund
WHO: World Health Organisation
