# Supplementary material for: Protocol for a systematic review of the effects of interventions for vaccine stock management
Source: Syst Rev. 2019 Jan 8;8:14. doi: 10.1186/s13643-018-0922-3 (PMC6323754; doi:10.1186/s13643-018-0922-3)
Supplement: Supplementary file 1 — PRISMA P checklist for the protocol. (DOCX 21 kb) [file 13643_2018_922_MOESM1_ESM.docx]

**Additional file 1**: PRISMA P checklist for the protocol

| **Section/topic** | **#** | **Checklist item** | **Information reported** | | **Page numbers\(s)** |
| --- | --- | --- | --- | --- | --- |
|  |  |  | **Yes** | **No** |  |
| **ADMINISTRATIVE INFORMATION** | | | | | |
| **Title** | | | | | |
| **Identification** | **1a** | **Identify the report as a protocol of a systematic review** |  |  | **1** |
| **Update** | **1b** | **If the protocol is for an update of a previous systematic review, identify as such** |  |  |  |
| **Registration** | **2** | **If registered, provide the name of the registry (e.g., PROSPERO) and registration number in the Abstract** |  |  | **1** |
| **Authors** | | | | | |
| **Contact** | **3a** | **Provide name, institutional affiliation, and e-mail address of all protocol authors; provide physical mailing address of corresponding author** |  |  | **1** |
| **Contributions** | **3b** | **Describe contributions of protocol authors and identify the guarantor of the review** |  |  | **3** |
| **Amendments** | **4** | **If the protocol represents an amendment of a previously completed or published protocol, identify as such and list changes; otherwise, state plan for documenting important protocol amendments** |  |  |  |
| **Support** | | | | | |
| **Sources** | **5a** | **Indicate sources of financial or other support for the review** |  |  | **3** |
| **Sponsor** | **5b** | **Provide name for the review funder and/or sponsor** |  |  | **3** |
| **Role of sponsor/funder** | **5c** | **Describe roles of funder(s), sponsor(s), and/or institution(s), if any, in developing the protocol** |  |  |  |
| **INTRODUCTION** | | | | | |
| **Rationale** | **6** | **Describe the rationale for the review in the context of what is already known** |  |  | **2** |
| **Objectives** | **7** | **Provide an explicit statement of the question(s) the review will address with reference to participants, interventions, comparators, and outcomes (PICO)** |  |  | **2** |
| **METHODS** | | | | | |
| **Eligibility criteria** | **8** | **Specify the study characteristics (e.g., PICO, study design, setting, time frame) and report characteristics (e.g., years considered, language, publication status) to be used as criteria for eligibility for the review** |  |  | **2** |
| **Information sources** | **9** | **Describe all intended information sources (e.g., electronic databases, contact with study authors, trial registers, or other grey literature sources) with planned dates of coverage** |  |  | **2** |
| **Search strategy** | **10** | **Present draft of search strategy to be used for at least one electronic database, including planned limits, such that it could be repeated** |  |  | **Appendix 1**  **3** |
| ***STUDY RECORDS*** | | | | | |
| **Data management** | **11a** | **Describe the mechanism(s) that will be used to manage records and data throughout the review** |  |  | **2** |
| **Selection process** | **11b** | **State the process that will be used for selecting studies (e.g., two independent reviewers) through each phase of the review (i.e., screening, eligibility, and inclusion in meta-analysis)** |  |  | **2** |
| **Data collection process** | **11c** | **Describe planned method of extracting data from reports (e.g., piloting forms, done independently, in duplicate), any processes for obtaining and confirming data from investigators** |  |  | **2** |
| **Data items** | **12** | **List and define all variables for which data will be sought (e.g., PICO items, funding sources), any pre-planned data assumptions and simplifications** |  |  | **2** |
| **Outcomes and prioritization** | **13** | **List and define all outcomes for which data will be sought, including prioritization of main and additional outcomes, with rationale** |  |  | **2** |
| **Risk of bias in individual studies** | **14** | **Describe anticipated methods for assessing risk of bias of individual studies, including whether this will be done at the outcome or study level, or both; state how this information will be used in data synthesis** |  |  | **2** |
| ***DATA*** | | | | | |
| **Synthesis** | **15a** | **Describe criteria under which study data will be quantitatively synthesized** |  |  | **2** |
|  | **15b** | **If data are appropriate for quantitative synthesis, describe planned summary measures, methods of handling data, and methods of combining data from studies, including any planned exploration of consistency (e.g., *I* ^2^, Kendall’s tau)** |  |  | **2-3** |
|  | **15c** | **Describe any proposed additional analyses (e.g., sensitivity or subgroup analyses, meta-regression)** |  |  | **3** |
|  | **15d** | **If quantitative synthesis is not appropriate, describe the type of summary planned** |  |  | **2** |
| **Meta-bias(es)** | **16** | **Specify any planned assessment of meta-bias(es) (e.g., publication bias across studies, selective reporting within studies)** |  |  | **3** |
| **Confidence in cumulative evidence** | **17** | **Describe how the strength of the body of evidence will be assessed (e.g., GRADE)** |  |  | **2** |
